# The Diagnostic Value of Soluble ST2 in Heart Failure: A Meta-Analysis

**DOI:** 10.3389/fcvm.2021.685904

**Published:** 2021-07-12

**Authors:** Chaojun Yang, Zhixing Fan, Jinchun Wu, Jing Zhang, Wei Zhang, Jian Yang, Jun Yang

**Affiliations:** ^1^Central Laboratory, Department of Cardiology, The First College of Clinical Medical Science, China Three Gorges University and Yichang Central People's Hospital, Yichang, China; ^2^Department of Cardiology, Qinghai Provincial People's Hospital, Xining, China; ^3^Department of Cardiology, Renmin Hospital of Wuhan University, Wuhan, China; ^4^Department of Cardiology, The People's Hospital of Three Gorges University and The First People' s Hospital of Yichang, Yichang, China

**Keywords:** soluble suppression of tumorigenicity, heart failure, diagnostic value, sensitivity, specificity, meta-analysis

## Abstract

**Objective:** The diagnostic performance of soluble suppression of tumorigenicity (sST2) in heart failure (HF) had been investigated in multiple studies, but the results were inconsistent. This meta-analysis evaluated the diagnostic value of sST2 in HF.

**Methods:** Pubmed, Web of Science, Embase, and Cochrane Library databases were searched until March 2021. Cohort studies or case-control studies relevant to the diagnostic value of sST2 in HF were screened, and true positive (TP), false positive (FP), false negative (FN), and true negative (TN) data were extracted for calculating sensitivity, specificity, positive likelihood ratio (PLR), negative likelihood ratio (NLR), diagnostic odds ratio (DOR), and area under the curve (AUC). The quality of the included studies was evaluated using the Quality Assessment of Diagnostic Accuracy Studies (QUADAS), the threshold effect was determined by calculating Spearman correlation coefficients and summary receiver operating characteristic (SROC) curve patterns, the heterogeneity was evaluated using the *I*^2^ statistic and the Galbraith radial plot, and sensitivity analysis was also performed. Deeks' test was used to assess publication bias.

**Results:** A total of 11 studies from 10 articles were included in this meta-analysis. The Spearman correlation coefficient was 0.114, *p* = 0.739, and the SROC curve did not show a “shoulder-arm” shape, which suggests that there was no threshold effect, but study heterogeneity existed because of non-threshold effects. The combined sensitivity was 0.72 [95% confidence interval (CI): 0.65–0.78], specificity was 0.65 (95% CI: 0.45–0.81), PLR was 1.75 (95% CI: 1.33–2.31), NLR was 0.48 (95% CI: 0.37–0.63), DOR was 3.63 (95% CI: 2.29–5.74), and AUC was 0.75. The Deeks' test suggested no significant publication bias in the included studies (*P* = 0.94).

**Conclusion:** sST has some diagnostic value in HF, but this should be further evaluated in additional studies with rigorous design and high homogeneity.

## Introduction

Heart failure (HF) is a clinical syndrome of cardiac blood flow impairment caused by ventricular systolic or diastolic insufficiency. It is a global health concern with high morbidity and mortality and has seriously endangered human health (1). Currently, HF is diagnosed based on clinical symptoms, medical history, echocardiography, B-type natriuretic peptide (BNP), and N-terminal (NT)-proBNP (2). However, because of the atypical symptoms and signs of HF, the ancillary tests such as echocardiography and invasive hemodynamics are often limited by factors such as medical condition, and BNP or NT-proBNP levels are easily affected by age, sex, body size, and renal function, which makes the diagnosis and management of HF still a clinical challenge (3). Simple, sensitive, and specific techniques are required to assist in the diagnosis of HF, and HF-related biological markers are the current focus of HF diagnosis (4). Soluble suppression of tumorigenicity 2 (sST2), a marker associated with cardiomyocyte traction, is a potential pathophysiological mediator of myocardial hypertrophy and myocardial fibrosis and an important biomarker of HF (5). Several trials have now confirmed that sST2 levels are significantly elevated in patients with HF and that the elevated levels of sST2 correlate significantly with the degree of HF (6, 7). In recent years, more studies have been reported on the diagnosis of HF using sST2, but the results of these studies vary significantly. In this study, we intend to systematically evaluate the diagnostic value of sST2 in HF using meta-analysis.

## Data and Methods

### Literature Search Strategy

For English databases Pubmed, Web of Science, Embase, and Cochrane Library, Heart failure, ST2, and diagnostic test were searched as the key words by the combination of medical subject headings (MeSH) and entry term. The literature search start date was not restricted, and the search end date was March 2021. The search language was only English. The following search strategy was used for pubmed and modified to suit other databases (the detailed retrieval strategy of other databases in Supplementary Documents):

#1 heart failure[MeSH Terms]
#2 ((((((((((((((Cardiac Failure[Title/Abstract]) OR (Heart Decompensation[Title/Abstract])) OR (Decompensation, Heart[Title/Abstract])) OR (Heart Failure, Right-Sided[Title/Abstract])) OR (Heart Failure, Right Sided[Title/Abstract])) OR (Right-Sided Heart Failure[Title/Abstract])) OR (Right Sided Heart Failure[Title/Abstract])) OR (Myocardial Failure[Title/Abstract])) OR (Congestive Heart Failure[Title/Abstract])) OR (Heart Failure, Congestive[Title/Abstract])) OR (Heart Failure, Left-Sided[Title/Abstract])) OR (Heart Failure, Left Sided[Title/Abstract])) OR (Left-Sided Heart Failure[Title/Abstract])) OR (Left Sided Heart Failure[Title/Abstract]))) OR (HF)
#3 #1 OR #2
#4 ((((((Soluble suppression of tumorigenicity 2[Title/Abstract]) OR (Soluble suppression of tumorigenicity-2[Title/Abstract])) OR (suppression of tumorigenicity 2[Title/Abstract])) OR (suppression of tumorigenicity-2[Title/Abstract])) OR (sST2[Title/Abstract])) OR (ST2[Title/Abstract])) OR (soluble ST2[Title/Abstract])
#5 “sensitiv^*^”[Title/Abstract] OR “sensitivity and specificity”[MeSH Terms] OR (“predictive”[Title/Abstract] AND “value^*^”[Title/Abstract]) OR (“predictive value of tests”[MeSH Terms] OR (“predictive”[All Fields] AND “value”[All Fields] AND “tests”[All Fields]) OR “predictive value of tests”[All Fields]) OR “accuracy^*^”[Title/Abstract]
#6 #3 AND #4 AND #5.

### Literature Inclusion and Exclusion Criteria

Literature inclusion criteria: (1) cohort studies or case-control studies investigating sST2 for the diagnosis of HF; (2) valid data available in the literature for the calculation of true positives (TPs), false positives (FPs), false negatives (FNs), and true negatives (TNs) to obtain information for a four-grid table; and (3) high quality studies using quality evaluation (see below). Exclusion criteria: (1) reviews, conference papers, and letters; (2) literature that cannot provide valid data for a four-grid table; (3) literature with duplicate data; (4) literature reporting results from animals or cellular models; (5) literature with too small a sample size (*n* <100); (6) literature of low quality using quality evaluation. This systematic evaluation was performed by two authors who independently judged whether the retrieved literature could be included in the study, and the third author made an independent judgment whether to include it in case of disagreement.

### Literature Quality Evaluation Criteria

The quality assessment of diagnostic accuracy studies (QUADAS) tool provided by the Cochrane Collaboration system was used to evaluate the quality of the literature. The QUADAS tool evaluates the four biases in terms of case selection, trials to be evaluated, gold standard, and flow, and it evaluates the quality of the literature by assessing 11 landmark questions and three types of clinical applicability questions. The 11 landmark issues were evaluated as “Yes” for clear fit, “Unclear” for unclear, and “No” for not meeting the conditions; the four biases were evaluated as “High risk” for clear bias, “Unclear” for unclear bias, and “Low risk” for no clear bias; and the three types of clinical applicability were evaluated as “High concern” for good matches, “Unclear” for unclear matches, and “Low concern” for poor matches. For each included study, two authors evaluated the quality independently, and the third author made an independent judgment in case of disagreement.

### Data Extraction

The extracted information included the basic information of the study and the four-grid table information. The basic information included authors, year of publication, country, sample size, mean age, sST2 detection method, sST2 cut-off, HF diagnostic criteria, HF type, control population, and study type. TP, FP, TN, and FN data were extracted from the included studies, and data that could not be extracted directly could be obtained by data transformation or by contacting the authors.

### Statistical Methods

Statistical analysis of the data was performed using Stata 15 and Meta-Disc (version 14.0) software. First, threshold effects were determined using Spearman correlation coefficient and the pattern of the summary receiver operating characteristic cure (SROC) curve. Then, the combined effect indicators—sensitivity, specificity, positive likelihood ratio (PLR), negative likelihood ratio (NLR), diagnostic odds ratio (DOR), and area under the curve (AUC) of SROC—were calculated. Heterogeneity was tested with the chi-square test using the *I*^2^ of Q statistic, and *I*^2^ <50% or *P* > 0.05 indicated no significant heterogeneity among studies, and the effect indicators were combined using the fixed effect model (FEM); *I*^2^ > 50% or *P* < 0.05 indicated a significant heterogeneity among studies, so the effect indicators were combined using the randomized effect model (REM), and heterogeneity analysis and sensitivity analysis were conducted. The Deeks' test was used to assess publication bias. *P* < 0.05 was considered a statistically significant difference.

## Results

### Literature Search Results

**Figure 1:**
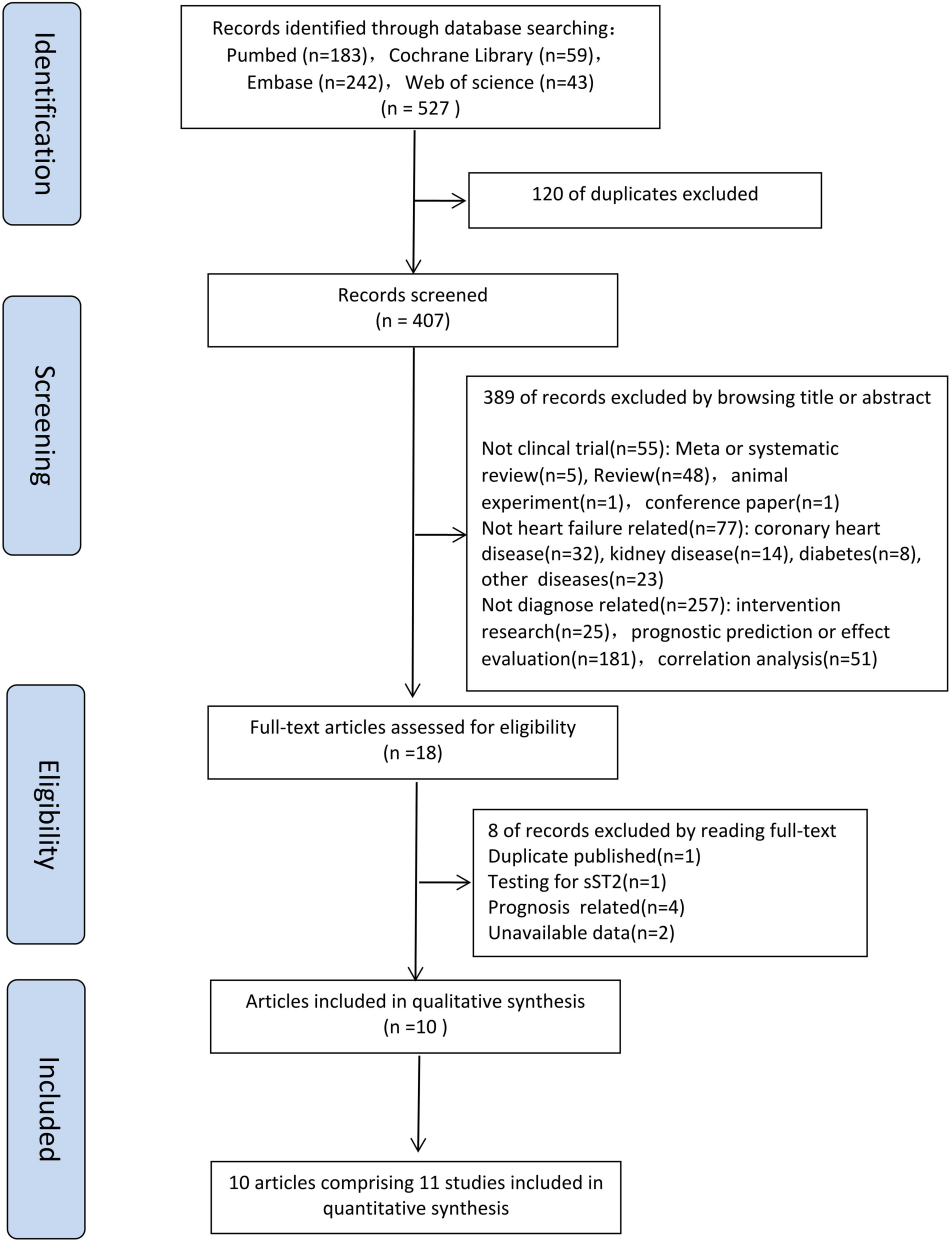
Flow diagram for study selection.

Five hundred and twenty-seven articles were obtained by searching with the proposed input, and a total of 407 articles were retrieved after removing duplicates. By reading the titles and abstracts, 389 articles were initially excluded (55 were not clincal trial; 77 were not heart failure related; 257 were not diagnose related) according to the inclusion and exclusion criteria. A total of 18 articles was investigated, and eight of them were excluded by reading full-text. For the eight excluded articles, one was duplicate publication, one was testing for sST2, four were prognosis related and two with no access to the four-grid table information. Finally, 10 articles with 11 studies were included in the meta-analysis (8–17) (Figure 1).

### Basic Characteristics of the Included Literature

**Figure 2:**
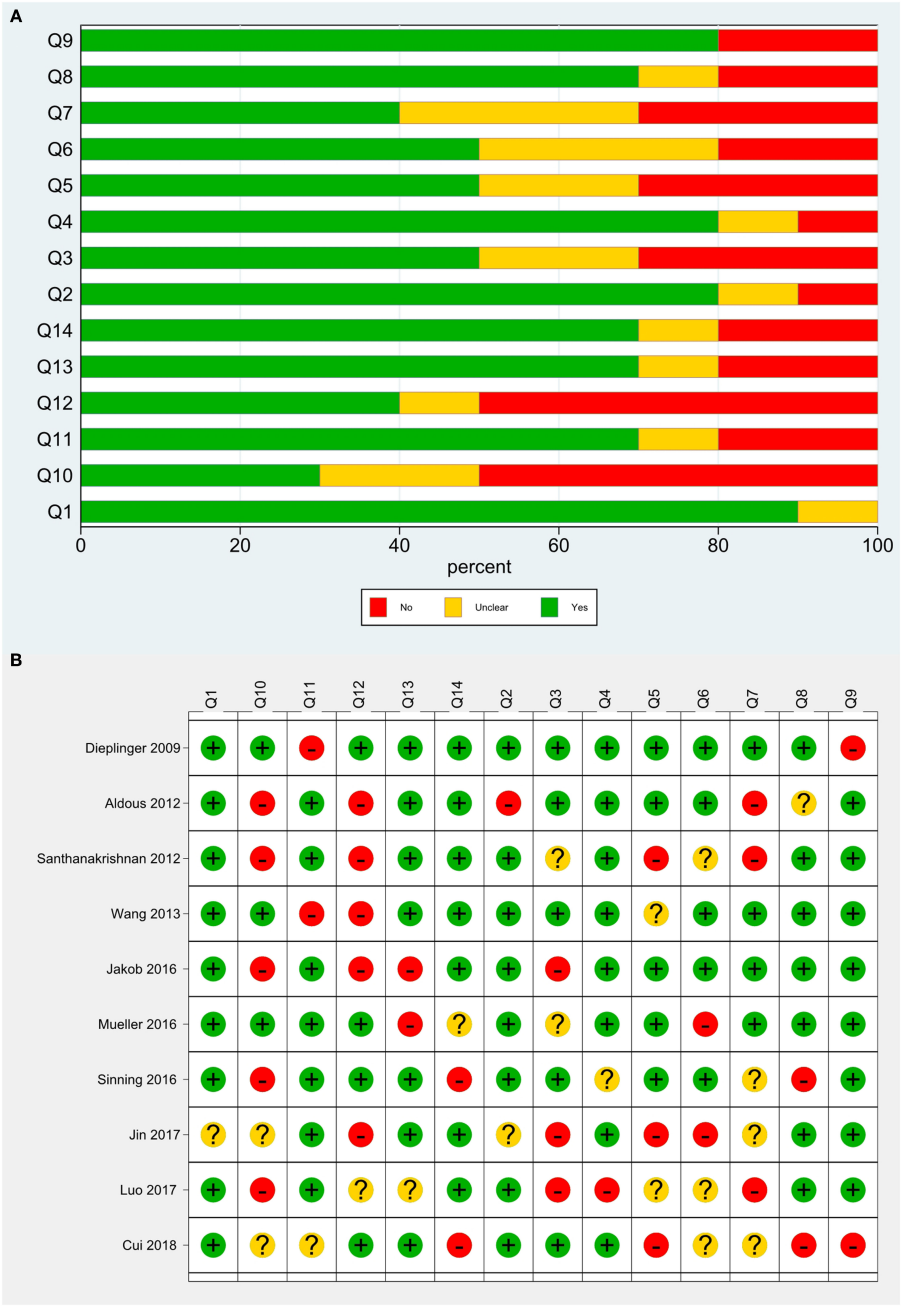
Quality evaluation of the included studies. **(A)** Review authors' judgments presented as percentages for the included studies; **(B)** Review authors' judgements for each included study.

A total of 11 studies were included. Santhanakrishnan et al. (9) divided HF patients into HF with preserved ejection fraction (HFPEF) and HF with reduced ejection fraction (HFREF) and studied them separately, and thus this literature was considered as two studies. The basic information of the included studies is shown in Table 1. The total sample size of 8,361 patients was included, involving cases from Australia, New Zealand, Singapore, the United Kingdom, Germany, and China. Ten studies investigated middle-aged and elderly populations, and one study focused on children. There were five cohort studies and six case-control studies. sST2 was detected using enzyme-linked immunosorbent assays (ELISAs), and sST2 kits were available from five manufacturers, including MBL, Presage^TM^, and R&D. Regarding the type of HF, four studies included patients with HFPEF, one included patients with HFREF, and the other six studies did not distinguish between reduced and preserved ejection fractions. The control population included people with dyspnea unrelated to HF, children with hypertension unrelated to HF, healthy populations, and community populations. TP, FP, FN, and TN data were extracted from each study for the meta-analysis (Table 2), and the quality evaluation of the included studies is shown in Figure 2.

**Table 1:** Characteristics of the studies included in this meta-analysis.

**No**	**References**	**Year**	**Country**	**Sample size**	**Male**	**Average age (year)**	**sST2 ELISAS kit source**	**Cut-off value**	**HF diagnostic criteria**	**Type of HF**	**Medical history of HF**	**Treatment history**	**Characteristics of controls**	**Type of research**
						**HF/no HF**								
1	Dieplinger et al. (8)	2009	Australia	251	234	72.82	MBL	121 ng/L	Framingham	HF	Arterial hypertension, diabetes mellitus	ACEI, ARB, calcium antagonist, β-blockers, digitalis, diuretics, amiodarone	ED patients with dyspnea	Cohort
2	Aldous et al. (9)	2012	New Zealand	995	591	66.00	-	34.3 U/mL	Chest radiograph evidence of pulmonary edema or symptoms of HF with raised BNP	HF	Ischemic heart disease, lung disease, stroke, Hypertension, dyslipidemia	-	ED patients with ischemic type pain	Cohort
3	Santhanakrishnan et al. (10)	2012	Singapore	100	52	66.00	Presage^TM^	26.47 ng/mL	Framingham	HFPEF	Diabetes mellitus, hypertension, coronary artery disease, stroke	ACEI/ARB, Spironolactone, β-blocker, diuretics, digoxin, statin, aspirin,	Community adults	Case-Control
4	Santhanakrishnan et al. (10)	2012	Singapore	101	66	60.98	Presage^TM^	30.32 ng/mL	Framingham	HFREF			Community adults	Case-Control
5	Wang et al. (11)	2013	Taiwan	107	57	65.08	R&D	13.5 ng/mL	Framingham	HFPEF	Diabetes, dyslipidemia, coronary artery disease, atrial fibrillation	Aspirin, nitrates, calcium channel blockers, ACEI/ARB, β-Blockers, diuretics, statins, antiarrythmic agents	Outpatients with hypertension	Cohort
6	Jakob et al. (12)	2016	Austria and UK	203		7.5	Presage^TM^	44.4 pg/mL	Presence of HF symptoms and abnormal ventricular systolic function	HF	Dilated cardiomyopathy, functional single ventricle, pulmonary/right-sided obstruction, aortic/left-sided obstruction, ventricular septal defect, tetralogy of fallot, atrioventricular septal defect, patent arterial duct, hypertrophic cardiomyopathy, restrictive cardiomyopathy, atrial septal defect, mixed lesion/other	-	children without heart disease undergoing phlebotomy prior to an elective procedure	Case-Control
7	Mueller et al. (13)	2016	Austria	251	234	76/69	Presage^TM^	26.5 ng/mL	Framingham	HF	Arterial hypertension, diabetes mellitus, atrial fibrillation, coronary artery disease	ACEI/ARB, calcium antagonists, β-blockers, digitalis, diuretics, amiodarone	dyspnoea attributed to other reasons	Cohort
8	Sinning et al. (14)	2016	Germany	4,972	2,526	67/55	Presage^TM^	-	NYHA	HF	Diabetes, hypertension, dyslipidemi	-	Recruitment with no HF	Cohort
9	Jin et al. (15)	2017	China	303	200	61.89/60.31	Shanghai Research Institute for Enzyme-linked Biology	-	ESC Guidelines	HF	-	-	Healthy people	Case-Control
10	Luo et al. (16)	2017	China	876	460	67.49/65.93	–	0.159 μg/L	China Guidelines	HFPEF	Coronary heart disease, diabetes mellitus, hypertension, fatty liver, carotid plaque, gout	Antiplatelet drugs, ACEI/ARB, β-blockers, trimetazidine, diuretics, statins, digitalis	healthy individuals	Case-Control
11	Cui et al. (17)	2018	China	202	135	73/67	Shanghai Qiyi Biological Co.	68.6 pg/mL	ESC Guidelines	HFPEF	Hypertension, diabetes mellitus, coronary heart disease, Atrial fibrillation	β-blocker, ARB, dioxin, aldosterone antagonist, statin	Health examiner	Case-Control

**Table 2:** Main findings of the included studies.

**References**	**TP**	**FP**	**FN**	**TN**	**SEN**	**SPE**
Dieplinger et al. (8)	123	89	14	25	0.90	0.22
Aldous et al. (9)	25	196	9	765	0.74	0.80
Santhanakrishnan et al. (10)	35	26	15	24	0.70	0.48
Santhanakrishnan et al. (10)	35	16	16	34	0.69	0.68
Wang et al. (11)	50	10	18	29	0.74	0.74
Jakob et al. (12)	65	39	49	50	0.57	0.56
Mueller et al. (13)	104	58	33	56	0.76	0.49
Sinning et al. (14)	81	2,882	27	1,982	0.75	0.41
Jin et al. (15)	154	0	43	106	0.78	1.00
Luo et al. (16)	267	166	109	334	0.71	0.67
Cui et al. (17)	83	13	89	17	0.48	0.57

*TP, True positive; FP, False positive; FN, False negative; TN, True negative; SEN, Sensitivity; SPE, Specificity*.

### Threshold Effect Analysis

Meta-disc analysis showed that the Spearman correlation coefficient between the log of sensitivity and the log of (1-specificity) was 0.114, *P* = 0.739, and the SROC curve showed no “shoulder-arm” pattern, which suggests that there was no threshold effect in this study.

### Diagnostic Value of sST2 in Patients With HF

**Figure 3:**
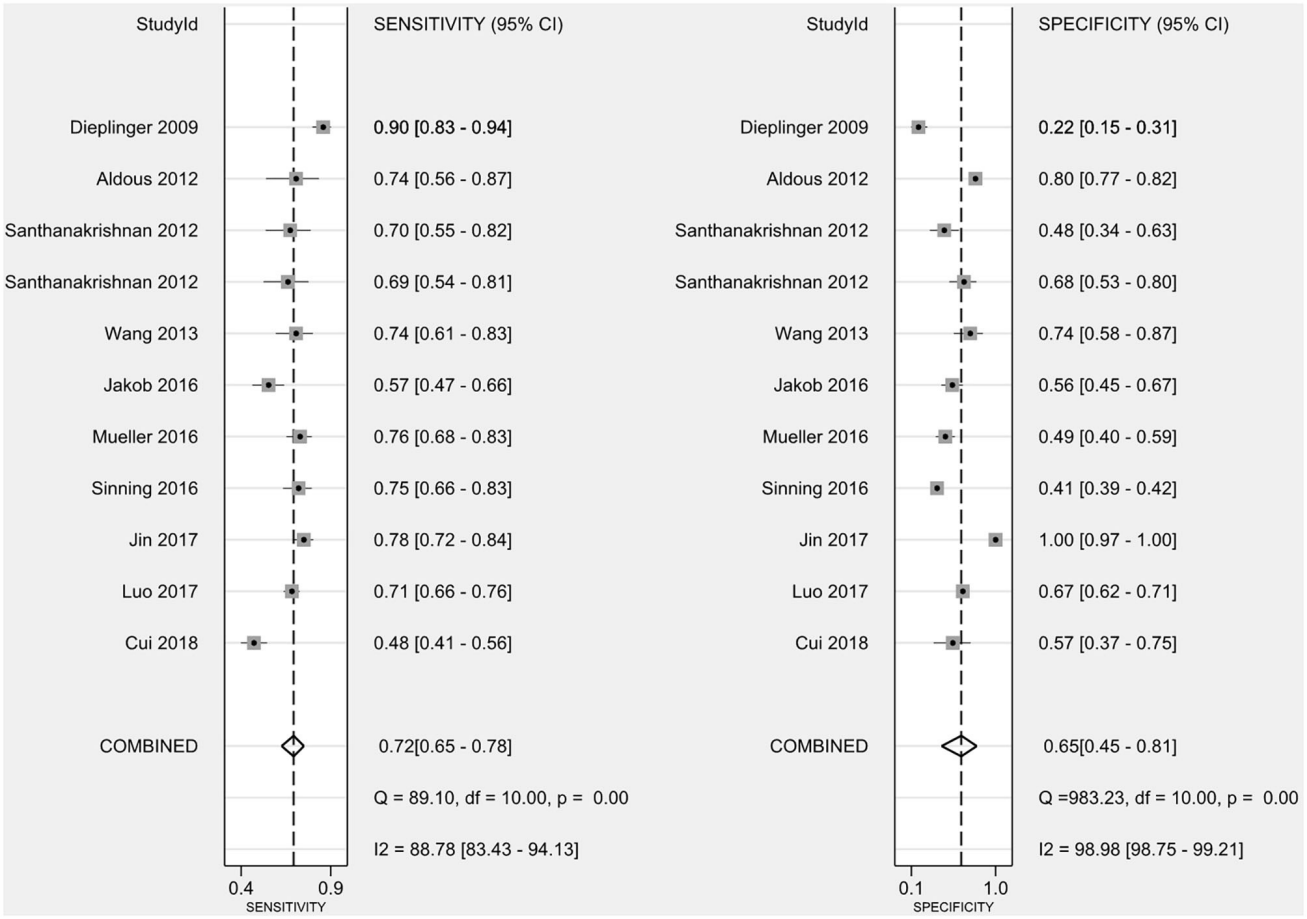
The combined sensitivity and specificity of sST2 for the diagnosis of HF. sST2, soluble suppression of tumorigenicity; HF, heart failure.

**Figure 4:**
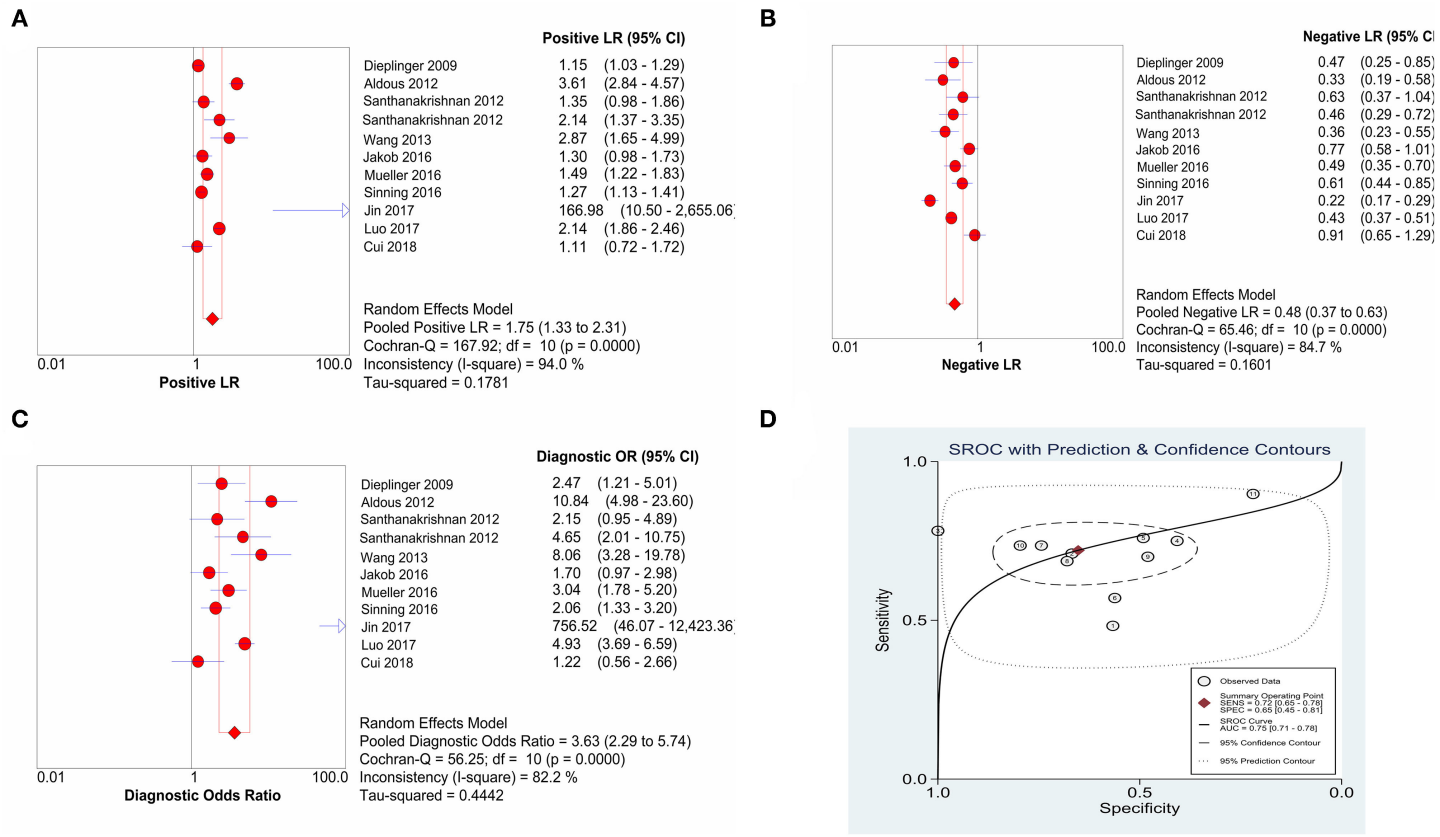
The forest plots of **(A)** PLR, **(B)** NLR, **(C)** DOR, and **(D)** AUC of SROC. PLR, positive likelihood ratio; NLR, negative likelihood ratio; DOR, diagnostic odds ratio; AUC, area under curve; SROC, summary receiver operating characteristic.

The combined sensitivity of sST2 for the diagnosis of HF was 0.72 (95% confidence interval (CI): 0.65–0.78) (Figure 3), the combined specificity was 0.65 (95% CI: 0.45–0.81) (Figure 3), the combined PLR was 1.75 (95% CI: 1.33–2.31) (Figure 4A), the combined NLR was 0.48 (95% CI: 0.37–0.63) (Figure 4B), and the combined DOR was 3.63 (95% CI: 2.29–5.74) (Figure 4C). The AUC of the SROC curve was 0.75 (Figure 4D).

### Heterogeneity Analysis

**Figure 5:**
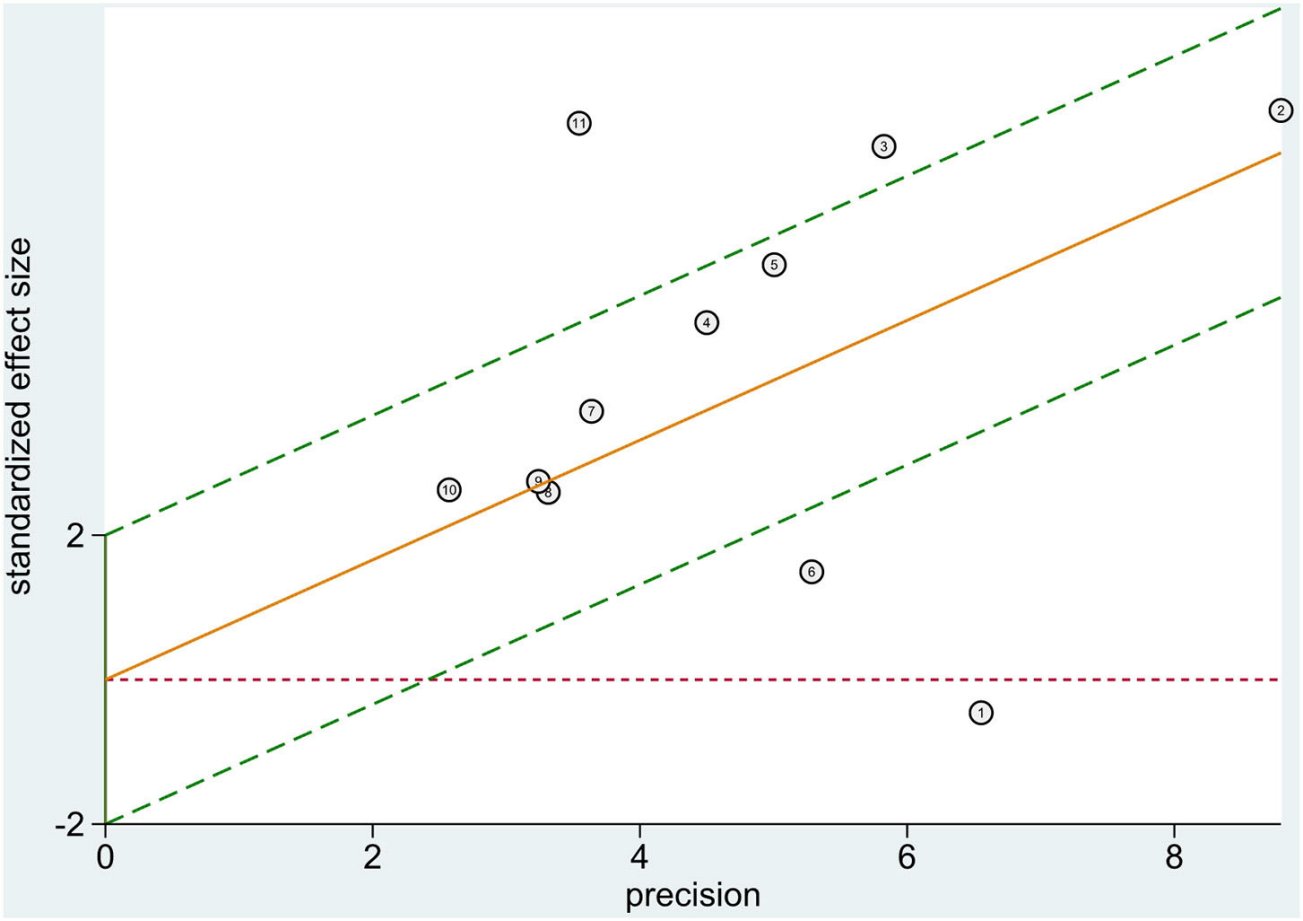
Heterogeneity analysis. Heterogeneity was evaluated by Galbraith radial plot.

Heterogeneity tests showed that *I*^2^ = 88.78% (*P* < 0.0001) for sensitivity, *I*^2^ = 98.98% (*P* < 0.0001) for specificity, *I*^2^ = 94.0% (*P* < 0.0001) for PLR, *I*^2^ = 84.7% (*P* < 0.0001) for NLR, and *I*^2^ = 82.2% (*P* < 0.0001) for DOR, which suggests the presence of heterogeneity unrelated to threshold effects in this study, so the effect sizes were combined using a randomized effect model and the source of heterogeneity was analyzed. The Galbraith radial plot (Figure 5) showed that four studies conducted by Dieplinger et al., Santhanakrishnan et al., Jakob et al., and Cui et al. were the sources of the heterogeneity.

### Sensitivity Analysis

**Figure 6:**
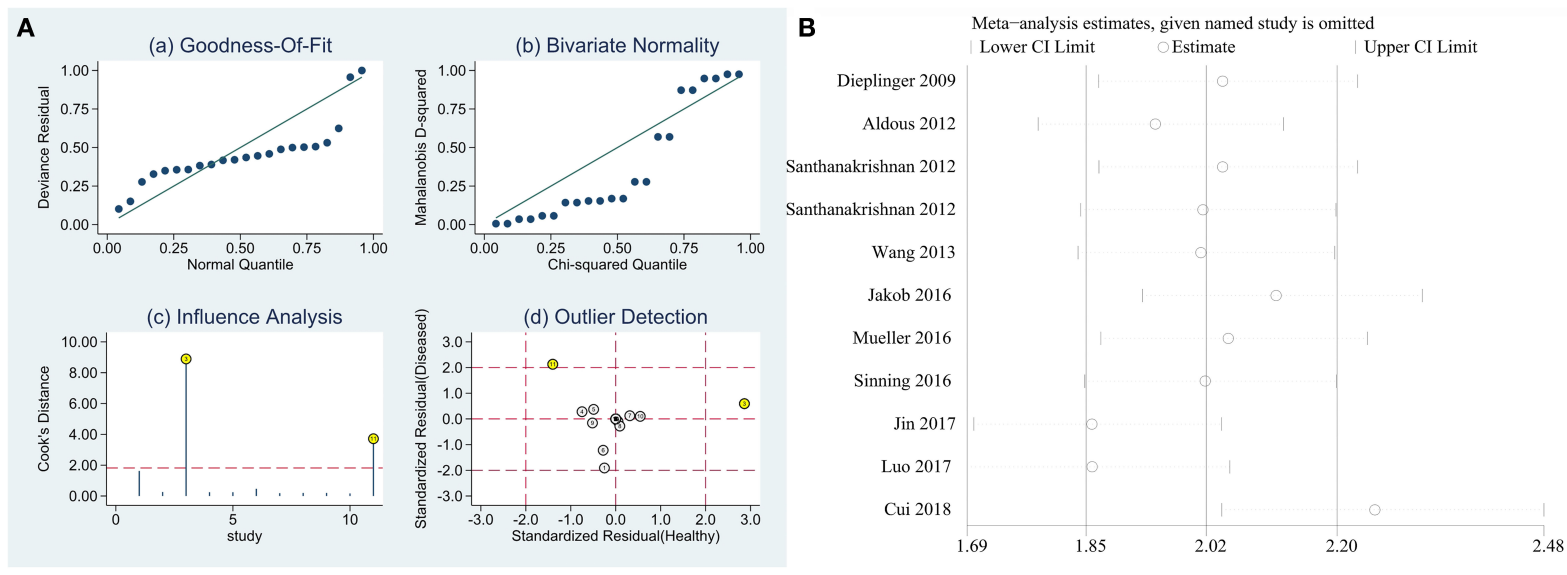
Sensitivity analysis diagram. **(A)** Sensitivity analysis, **(B)** Individual study exclusion.

Sensitivity analysis of the data from this study showed that the studies conducted by Santhanakrishnan et al. and Cui et al. had the most impact on the calculation of the results of this study (Figure 6A), while the other original studies had no impact on the calculation of the study results. Taken together, the results of this study were relatively stable. Sensitivity analysis of the impact of individual studies showed that the exclusion of the study conducted by Cui et al. had the most effect on the calculation of results in this meta-analysis (Figure 6B).

### Publication Bias

**Figure 7:**
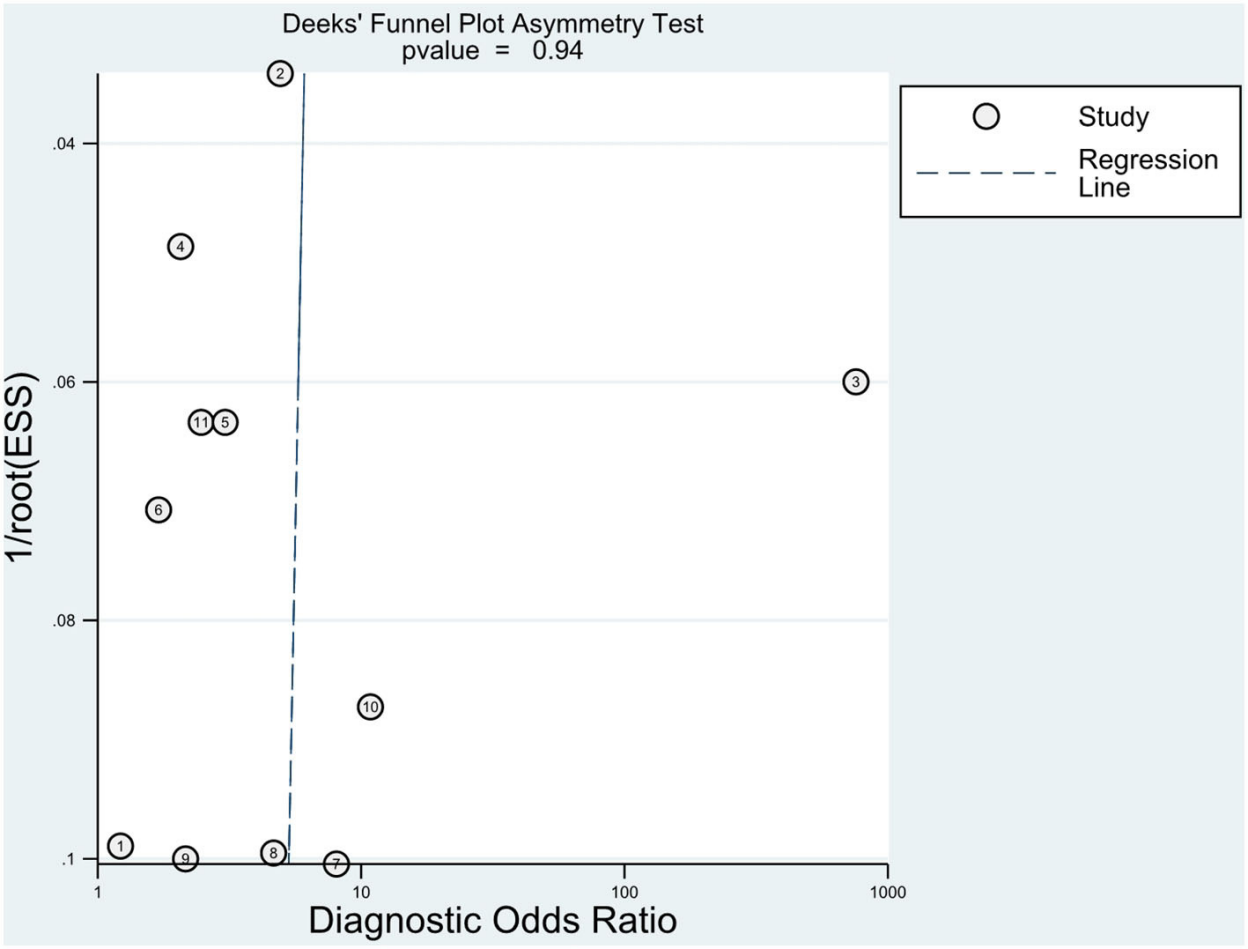
Publication bias. The Deek's test was established to evaluate publication bias.

The Deeks' test was performed using Stata software to assess publication bias (Figure 7); it showed a *P* = 0.94, which suggests that there was no significant publication bias in the included studies.

## Discussion

HF is a common outcome of multiple cardiovascular diseases. Cardiac overload and myocardial cell injury can lead to reduced cardiac function, which results in compensatory changes such as ventricular hypertrophy and chamber enlargement, as well as corresponding changes in cardiomyocytes, extracellular matrix, and collagen fiber networks followed by ventricular remodeling, leading to further deterioration of cardiac function (18). Multiple factors are involved in the progression of HF, such as myocardial necrosis, apoptosis, autophagy, fibrosis, oxidative stress, inflammatory response, and neurohumoral regulatory disorders, as well as changing levels in a series of biomarkers (19). The American College of Cardiology/American Heart Association/Heart Failure Society of America (ACC/AHA/HFSA) guidelines released in 2017 stated that BNP and NT-proBNP provide clear diagnostic value in patients with chronic HF (20). BNP or NT-proBNP has been used clinically as a routine test for HF, but it is susceptible to various factors such as age, sex, and disease condition. Among these biomarkers, the myocardial fibrosis marker sST2 is not affected by factors like age, sex, renal function, and weight (21). Meanwhile, the 2017 ACC/AHA/HFSA guidelines recommend measuring sST2 for risk stratification of patients with chronic HF (20). This suggests that sST2 has some value in the diagnosis and prognosis of HF.

ST2 is a member of the interleukin-1 (IL-1) receptor superfamily, which is encoded by the *ST2* gene in cardiomyocytes during myocardial stretch and under mechanical stress. The *ST2* gene is located on human chromosome 2q12 and can encode two isoforms of sST2 and the transmembrane receptor form of ST2 (ST2L). Interleukin-33 (IL-33) is a functional ligand for ST2, and the ST2/IL-33 signaling pathway exerts cardioprotective effects by activating ST2L receptors to reduce myocardial fibrosis, inhibit cardiomyocyte hypertrophy, and improve cardiac function, which does not require the sST2 receptor (22). During HF, the increased cardiac load exposes the myocardium to excessive stretch stimulation, and the overproduced sST2 can compete with ST2L for binding IL-33, abrogating the cardioprotective effect of the ST2/IL-33 signaling pathway; this leads to apoptosis, hypertrophy and fibrosis of cardiomyocytes, and further deterioration of cardiac function, aggravating the HF process (23). This suggests that sST2 plays an important role in the development of HF. Clinical studies on the diagnostic value of sST2 in HF have gradually increased in recent years (24). In this study, the clinical diagnostic value of sST2 in HF was evaluated using meta-analysis.

Meta-analysis showed that the combined sensitivity was 0.72, specificity was 0.65, DOR was 3.63, and AUC was 0.75, which indicates that sST2 has a good diagnostic value for HF. The meta-analysis conducted by Huang et al. (24) included 10 original studies, all of which were conducted before 2014 and published in either Chinese or English, and their combined sensitivity was 0.84, specificity was 0.74, DOR was 8.49, and AUC was 0.81. Both the sensitivity and specificity in this study were about 10% lower than those in the Huang et al. (24), which may be related to the inclusion of recent literature and more stringent quality screening performed in this study, but both meta-analyses had a high degree of heterogeneity. Diagnostic studies are generally more heterogeneous than other types of clinical studies because of a possible bias in case selection, trials to be evaluated, gold standards, and flow. This study showed that four studies, those conducted by Dieplinger et al., Santhanakrishnan et al., Jakob et al., and Cui et al. may be the source of heterogeneity in this meta-analysis, and the study conducted by Cui et al. had the most impact on the results of the meta-analysis. The analysis revealed that Dieplinger et al. used a sST2 kit from MBL, Santhanakrishnan et al. conducted a case-control study, Jakob et al. focused on children, and Cui et al. performed a case-control study on patients with HFPEF using a sST2 kit from Shanghai Qiyi Biological Co.; thus, the above-mentioned differences may have contributed to the large heterogeneity observed in this study. In addition, the heterogeneity of this study may have also been caused by the disease typing (different degrees of HF in different studies), the composition of the disease spectrum (the patient group may be combined with other diseases, and the control group includes patients with various cardiovascular diseases without HF), the diagnostic thresholds (the thresholds were not uniform among studies), and differences in the sST2 detection methods. Moreover, there was also heterogeneity because of mixed bias caused by the HF type, control population, sST2 kit, HF diagnostic criteria, study type, and other biases.

Although this meta-analysis included a relatively comprehensive literature search, there were still some limitations. First, the heterogeneity of the included studies was high, and the potential sources include HF type, control population, sST2 kit, HF diagnostic criteria, and study type, with possible heterogeneity between subgroups and from other sources. Second, most of the included studies were case-control studies, which could cause selection bias in the selection of study subjects and increase diagnostic sensitivity. Third, the diagnostic cut-off values of sST2 were not uniform, and the diagnostic cut-off values of sST2 varied among the 11 included studies, which may have been related to factors such as kits, test conditions, and sample-handling methods.

In general, sST2 has some diagnostic value for HF, but factors such as HF type, control population, sST2 kits, HF diagnostic criteria, and study type in the original studies may have affected its diagnostic value. Therefore, we still need to design prospective cohort studies with high quality, large sample sizes, uniform study populations, uniform control populations, and uniform test methods to further explore and validate the reliability of the results of this analysis; we also need to establish an accurate cut-off value for sST2 to provide clinical guidance for the diagnosis of HF.

## Data Availability Statement

The original contributions presented in the study are included in the article/Supplementary Material, further inquiries can be directed to the corresponding author/s.

## Author Contributions

This study was designed by ZF and JuY. CY and JiY contributed data to the paper. Statistical analysis and interpretation of data were performed by JiY, JZ, WZ, and JW. All authors were involved in drafting and revision of the manuscript for important intellectual content and approved the final version to be published.

## Conflict of Interest

The authors declare that the research was conducted in the absence of any commercial or financial relationships that could be construed as a potential conflict of interest.

## Supplementary Material

Supplementary Document 1 Literature searches and search strategy.

Supplementary Table 1 Included and excluded reference.
