# Supplementary material for: The Diagnostic Value of Soluble ST2 in Heart Failure: A Meta-Analysis
Source: Front Cardiovasc Med. 2021 Jul 12;8:685904. doi: 10.3389/fcvm.2021.685904 (PMC8315235; doi:10.3389/fcvm.2021.685904)

**Pubmed（n=183）**

#1 heart failure[MeSH Terms]

#2 ((((((((((((((Cardiac Failure[Title/Abstract]) OR (Heart Decompensation[Title/Abstract])) OR (Decompensation, Heart[Title/Abstract])) OR (Heart Failure, Right-Sided[Title/Abstract])) OR (Heart Failure, Right Sided[Title/Abstract])) OR (Right-Sided Heart Failure[Title/Abstract])) OR (Right Sided Heart Failure[Title/Abstract])) OR (Myocardial Failure[Title/Abstract])) OR (Congestive Heart Failure[Title/Abstract])) OR (Heart Failure, Congestive[Title/Abstract])) OR (Heart Failure, Left-Sided[Title/Abstract])) OR (Heart Failure, Left Sided[Title/Abstract])) OR (Left-Sided Heart Failure[Title/Abstract])) OR (Left Sided Heart Failure[Title/Abstract]))) OR (HF)

#3 #1 OR #2

#4 ((((((Soluble suppression of tumorigenicity 2[Title/Abstract]) OR (Soluble suppression of tumorigenicity-2[Title/Abstract])) OR (suppression of tumorigenicity 2[Title/Abstract])) OR (suppression of tumorigenicity-2[Title/Abstract])) OR (sST2[Title/Abstract])) OR (ST2[Title/Abstract])) OR (soluble ST2[Title/Abstract])

#5 "sensitiv*"[Title/Abstract] OR "sensitivity and specificity"[MeSH Terms] OR ("predictive"[Title/Abstract] AND "value*"[Title/Abstract]) OR ("predictive value of tests"[MeSH Terms] OR ("predictive"[All Fields] AND "value"[All Fields] AND "tests"[All Fields]) OR "predictive value of tests"[All Fields]) OR "accuracy*"[Title/Abstract]

#6 #3 AND #4 AND #5


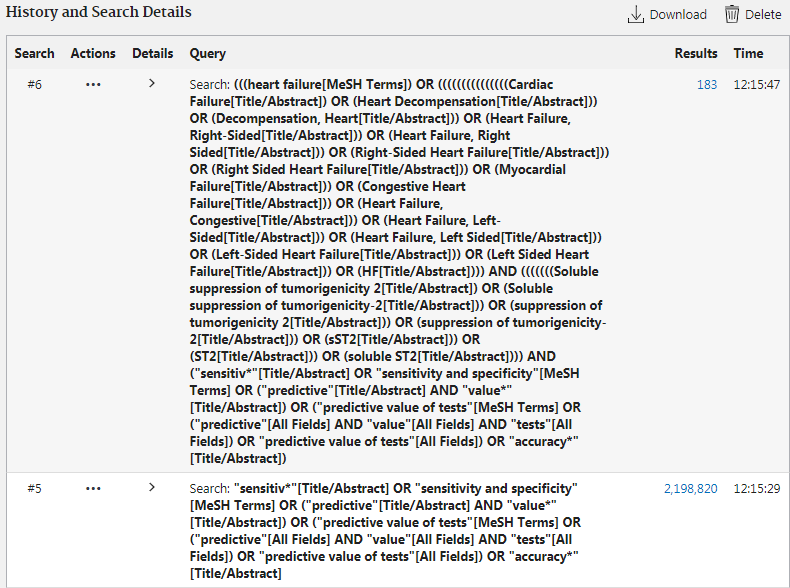


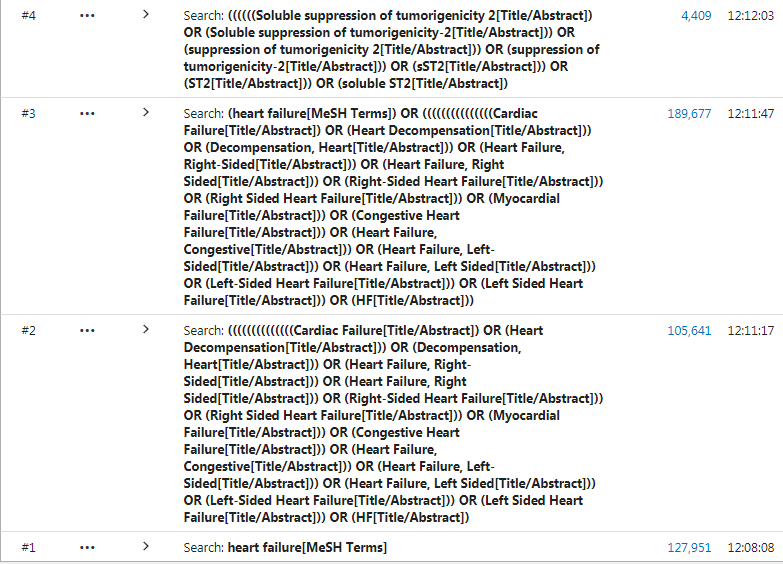


**Cochrane Library（n=59）**

#1 heart failure

#2 (Cardiac Failure):ab,ti,kw OR (Heart Decompensation):ab,ti,kw OR (Decompensation, Heart):ab,ti,kw OR (Heart Failure, Right-Sided):ab,ti,kw OR (Heart Failure, Right Sided):ab,ti,kw OR (Right-Sided Heart Failure):ab,ti,kw OR (Right Sided Heart Failure):ab,ti,kw OR (Myocardial Failure):ab,ti,kw OR (Congestive Heart Failure):ab,ti,kw OR (Heart Failure, Congestive):ab,ti,kw OR (Heart Failure, Left-Sided):ab,ti,kw OR (Heart Failure, Left Sided):ab,ti,kw OR (Left-Sided Heart Failure):ab,ti,kw OR (Left Sided Heart Failure):ab,ti,kw OR (HF):ab,ti,kw

#3 #1 OR #2

#4 (Soluble suppression of tumorigenicity 2):ab,ti,kw OR (Soluble suppression of tumorigenicity-2):ab,ti,kw OR (suppression of tumorigenicity 2):ab,ti,kw OR (suppression of tumorigenicity-2):ab,ti,kw OR (sST2):ab,ti,kw OR (ST2):ab,ti,kw OR (soluble ST2):ab,ti,kw

5# (sensitiv):ab,ti,kw OR (sensitivity and specificity):ab,ti,kw OR (predictive):ab,ti,kw OR (predictive value of tests):ab,ti,kw OR (accuracy):ab,ti,kw

#6 #3 AND #4 AND #5


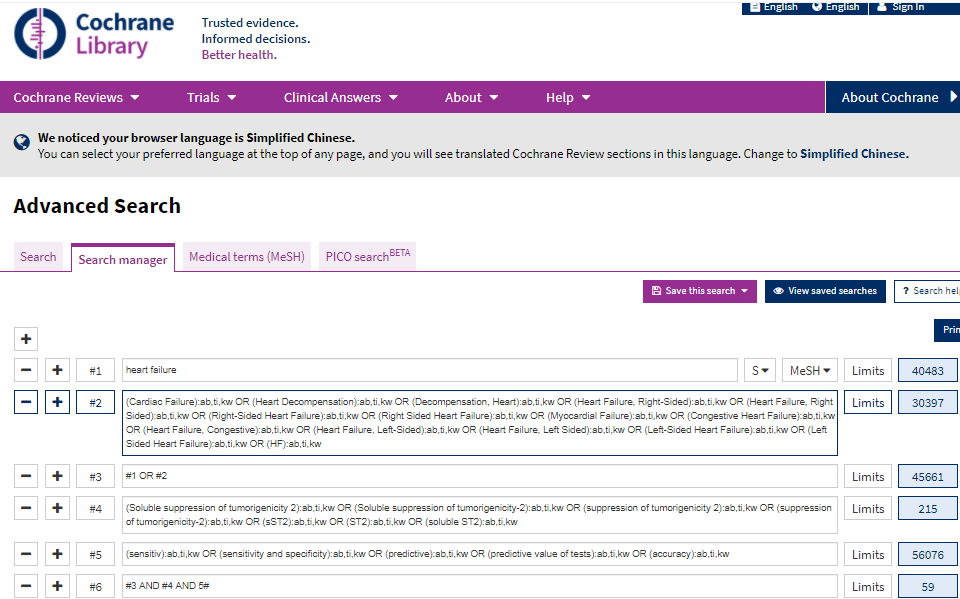


**Embase （n=242）**

#1 heart failure

#2 'Cardiac Failure':ab,ti OR 'Heart Decompensation':ab,ti OR 'Decompensation, Heart':ab,ti OR 'Heart Failure, Right-Sided':ab,ti OR 'Heart Failure, Right Sided':ab,ti OR 'Right-Sided Heart Failure':ab,ti OR 'Right Sided Heart Failure':ab,ti OR 'Myocardial Failure':ab,ti OR 'Congestive Heart Failure':ab,ti OR 'Heart Failure, Congestive':ab,ti OR 'Heart Failure, Left-Sided':ab,ti OR 'Heart Failure, Left Sided':ab,ti OR 'Left-Sided Heart Failure':ab,ti OR 'Left Sided Heart Failure':ab,ti OR 'HF':ab,ti

#3 #1 OR #2

#4 'Soluble suppression of tumorigenicity 2':ab,ti OR 'Soluble suppression of tumorigenicity-2':ab,ti OR 'suppression of tumorigenicity 2':ab,ti OR 'suppression of tumorigenicity-2':ab,ti OR 'sST2':ab,ti OR 'ST2':ab,ti OR 'soluble ST2':ab,ti

#5 'sensitiv':ab,ti OR 'sensitivity and specificity':ab,ti OR 'predictive':ab,ti OR 'predictive value of tests':ab,ti OR 'accuracy':ab,ti

#6 #3 AND #4 AND #5


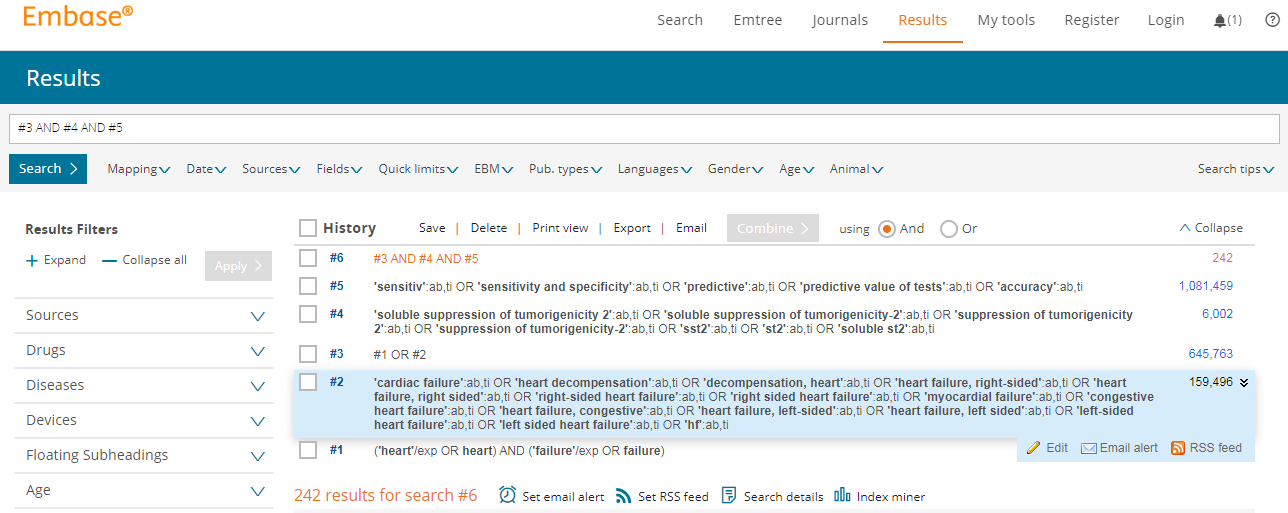


**Web of science （n=43）**

#1 heart failure or Cardiac Failure or Heart Decompensation or Decompensation, Heart or Heart Failure, Right-Sided or Heart Failure, Right Sided or Right-Sided Heart Failure or Right Sided Heart Failure or Myocardial Failure or Congestive Heart Failure or Heart Failure, Congestive or Heart Failure, Left-Sided or Heart Failure, Left Sided or Left-Sided Heart Failure or Left Sided Heart Failure or HF

#2 Soluble suppression of tumorigenicity 2 or Soluble suppression of tumorigenicity-2 or suppression of tumorigenicity 2 or suppression of tumorigenicity-2 or sST2 or ST2 or soluble ST2

#3 sensitiv or sensitivity and specificity or predictive or predictive value of test or accuracy

3 #1 AND #2 AND #3


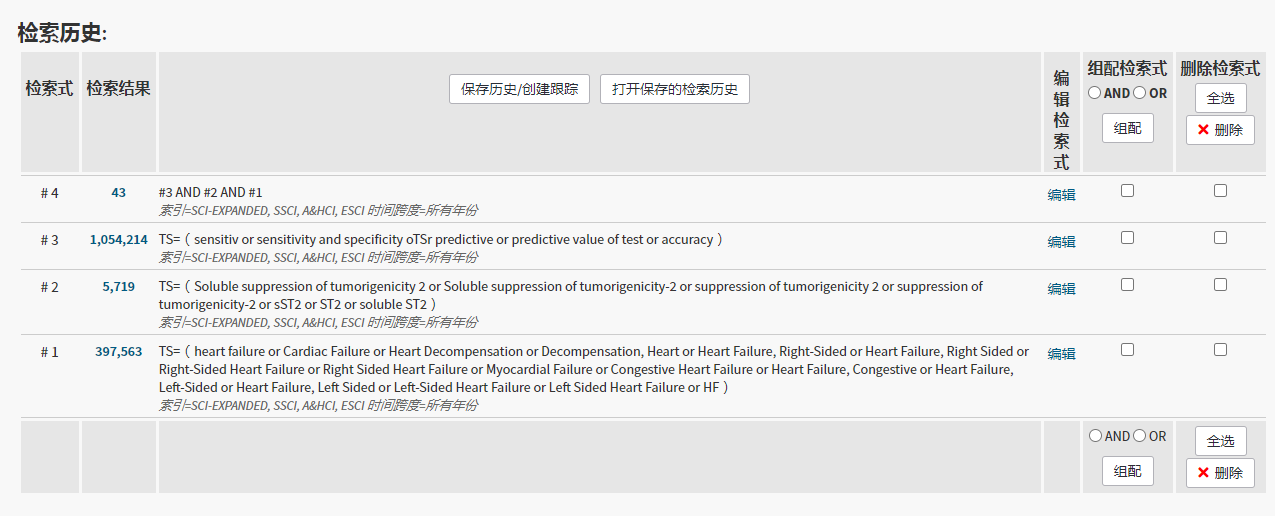

Supplement: Supplementary Document 1 — Literature searches and search strategy. [file Data_Sheet_1.doc]
